# Editorial: Current Perspectives of Antimicrobial Resistance in *Campylobacteraceae* and *Helicobacteraceae*

**DOI:** 10.3389/fcimb.2021.840456

**Published:** 2022-01-25

**Authors:** Arturo Levican, Guillermo Perez-Perez, Monica Oleastro, Heriberto Fernández, Susana Ferreira

**Affiliations:** ^1^ Sciences Faculty, School of Medical Technology, Pontificia Universidad Católica de Valparaíso, Valparaiso, Chile; ^2^ Langone Medical Center, New York University, New York City, NY, United States; ^3^ Instituto Nacional de Saúde Doutor Ricardo Jorge (INSA), Lisboa, Portugal; ^4^ Institute of Clinical Microbiology, Universidad Austral de Chile, Valdivia, Chile; ^5^ Centro de Investigação em Ciências da Saúde da Universidade da Beira Interior (CICS-UBI) Health Sciences Research Centre of University of Beira Interior, Covilhã, Portugal

**Keywords:** *Campylobacter*, *Helicobacter*, *Arcobacter*, biofilm, antimicrobial resistance, whole genome sequencing

*Campylobacter*, *Helicobacter*, and *Arcobacter* genera comprise major and emerging pathogens associated with gastroenteric diseases both in humans and animals. In addition, antibiotic-resistant populations have arisen in humans, as well as in animals, the environment, and food, with some species’ resistance being claimed as a major problem for the treatment of infectious diseases. Thus, it is imperative to surveil and improve knowledge of these bacteria resistance mechanisms to better fight them and use this knowledge in the development of new antimicrobial strategies.

In line with these issues, the present Research Topic in Frontiers in Cellular and Infection Microbiology includes a number of papers related to either novel treatments or assessment of antimicrobial resistance for *Campylobacter*, *Helicobacter*, and *Arcobacter*.

One of the challenges for the study of antimicrobial resistance arises from determining the influence of the planktonic and biofilm forms in drug efficacy. Biofilms could be present in infections as well as in equipment and processing surfaces. Furthermore, lower susceptibility to antimicrobial agents under biofilm forms has been observed compared to planktonic cells because it prevents access to bacteria and reduces the multiplication rate and bacterial metabolism. The intrinsic or plasmidial determinants of antibiotic resistance contribute to this profile and help ensure the survival of biofilm cells even under more aggressive antimicrobial treatment regimens (Hall and Mah, 2017). Aparecida et al. evaluated the effect of different classes of antimicrobials on the planktonic and biofilm forms of *Campylobacter jejuni* strains. The authors observed a link between antimicrobial resistance and the biomass density of the biofilm, except in the case of tetracycline. Interestingly, these authors also observed that colistin may be a new treatment approach and detected emerging resistance to meropenem that may be seen as alarming since it is a last therapeutic resource and a drug classified as critically important by the World Health Organization.

Developments in the field of bacterial whole genome sequencing (WGS) have made *in silico* antimicrobial resistance (AMR) a powerful tool to detect AMR genes and infer phenotypes. By using this approach, Rokney et al. report a high rate of tetracycline and quinolone resistance among Israeli *C. jejuni* strains and a low resistance rate to macrolides and aminoglycosides, revealing a high correlation rate between the presence of AMR determinants and phenotypic resistance. Moreover, Hänel et al. analyzed the antimicrobial susceptibility of the rarely isolated species *Aliarcobacter cibarius* and *Aliarcobacter thereius*, and provide detailed insights on their genotype and phylogeny using WGS. However, in both studies (Hänel et al. and Rokney et al.), some resistant phenotypes could not be predicted by the presence of AMR determinants. The authors outline that further work to improve the phenotypic prediction of AMR is crucial to the foreseen transition towards the use of WGS as the main methodology for foodborne isolate portrayal. Furthermore, there is a need to implement more robust species-oriented diagnostics. In this same line, Camorlinga-Ponce et al. studied trends of antibiotic resistance over a 20-year period in Mexican *H. pylori* strains. The authors compared susceptibility among strains from Mexican ethnically diverse populations by combining epsilometer tests and determining the occurrence of mutational patterns in specific genes by WGS. Increasing resistance to clarithromycin and levofloxacin was observed over 20 years in Mexican mestizo’ isolates, while resistance was lower for all antibiotics tested among native isolates. Although a good to moderate correlation was observed between phenotypic and genotypic approaches, the genetic methods for characterizing antibiotic resistance require further validation in each population.

Finally, innovative ways of coping with antibiotic resistance are essential for overcoming the challenges associated with animal and human health, but also food safety hazards. However, the use of some compounds, such as natural lipids may be limited by their physicochemical instability and toxicity. Considering this, Ribeiro et al. evaluated the use of bioactive vegetable oils against *C. jejuni*, employing nanotechnology (nanostructured lipid carriers). A formulation composed of ucuuba butter and olibanum essential oil was selected by its anti-*C. jejuni* activity and safety profile, and is proposed for the further analysis of this system in specific *in vivo* efficacy assays.

Together the articles published in this Research Topic bring insights on the resistance of *Campylobacter, Helicobacter*, and *Arcobacter species*, as well on the potential use of natural lipids as antimicrobial agents.

## Author Contributions

All authors listed have made a substantial, direct, and intellectual contribution to the work and approved it for publication.

## Conflict of Interest

The authors declare that the research was conducted in the absence of any commercial or financial relationships that could be construed as a potential conflict of interest.

## Publisher’s Note

All claims expressed in this article are solely those of the authors and do not necessarily represent those of their affiliated organizations, or those of the publisher, the editors and the reviewers. Any product that may be evaluated in this article, or claim that may be made by its manufacturer, is not guaranteed or endorsed by the publisher.
